# Sincere praise and flattery: reward value and association with the praise-seeking trait

**DOI:** 10.3389/fnhum.2023.985047

**Published:** 2023-02-15

**Authors:** Shotaro Fujiwara, Ryo Ishibashi, Azumi Tanabe-Ishibashi, Ryuta Kawashima, Motoaki Sugiura

**Affiliations:** ^1^School of Medicine, Tohoku University, Sendai, Japan; ^2^Smart-Aging Research Center, Tohoku University, Sendai, Japan; ^3^Institute of Development, Aging, and Cancer, Tohoku University, Sendai, Japan; ^4^International Research Institute of Disaster Science, Tohoku University, Sendai, Japan

**Keywords:** reward, praise, flattery, reliability, personality, fMRI, praise-seeking

## Abstract

Sincere praise reliably conveys positive or negative feedback, while flattery always conveys positive but unreliable feedback. These two praise types have not been compared in terms of communication effectiveness and individual preferences using neuroimaging. Through functional magnetic resonance imaging, we measured brain activity when healthy young participants received sincere praise or flattery after performing a visual search task. Higher activation was observed in the right nucleus accumbens during sincere praise than during flattery, and praise reliability correlated with posterior cingulate cortex activity, implying a rewarding effect of sincere praise. In line with this, sincere praise uniquely activated several cortical areas potentially involved in concern regarding others’ evaluations. A high praise-seeking tendency was associated with lower activation of the inferior parietal sulcus during sincere praise compared to flattery after poor task performance, potentially reflecting suppression of negative feedback to maintain self-esteem. In summary, the neural dynamics of the rewarding and socio-emotional effects of praise differed.

## 1 Introduction

Communication between people often includes praise for performance or status. Praise has rewarding (Lam et al., 2008) and socio-emotional effects [e.g., generating feelings of competence and happiness (Burnett and Mandel, 2010)]. We encounter two types of praise in daily conversation: sincere praise and flattery, which are differentiated on the basis of their relatedness to one’s performance, that is, feedback reliability (Fogg and Nass, 1997). Sincere praise involves positive and negative feedback based on performance or status (high reliability), while flattery involves positive feedback that does not necessarily reflect the true qualities or abilities of the praised individual (low reliability; thus superficial positive praise), including both accurate and inaccurate praise. Both sincere praise and flattery are rewarding for the receiver in different ways, but the different effects of the two types of praise are not obvious.

There are conflicting views on the rewarding effect of the two types of praise. Sincere praise is a social reward (Kohls et al., 2009), but the effect of flattery is controversial. The importance of reliability has long been recognized. Highly reliable text or feedback is more likely to change people’s behavior than less reliable types (Hovland and Weiss, 1951; Jacquot et al., 2015). Marketing research implies that flattery has negative effects on the speaker’s trustworthiness and a customer’s willingness to buy (Main et al., 2007). However, some studies have reported the positive effects of flattery. One study reported that flattery from a computer can be as effective as sincere praise in increasing the receiver’s willingness to perform a task (Fogg and Nass, 1997). In a marketing study, flattery was effective and persuasive, despite the ability of subjects to identify the insincerity of the praise (Chan and Sengupta, 2010).

“Praise-seeking” is an important personality trait that depends on sensitivity to the socio-emotional effects of praise. Some people prefer praise regardless of whether they deserve it, because positive words from others enhance the receiver’s status, intimacy (Leary and Kowalski, 1990), and self-esteem (Modigliani, 1968). The praise-seeking trait varies among individuals and has been measured using a standardized questionnaire (Kojima et al., 2003); its statistical reliability [Cronbach’s alpha coefficient = 0.83 (Kojima et al., 2003)] and statistical validity [the correlation with the Behavioral Activation System (BAS) score; *r* = 0.46, *p* < 0.01 (Kojima, 2018)] have been established. People with a high praise-seeking tendency experience positive emotions in response to positive feedback and negative emotions in response to negative feedback (Kojima et al., 2003). Flattery constitutes a type of positive feedback that is preferred by people with a high praise-seeking tendency, whereas sincere praise may be perceived as negative feedback when performance is low and is disliked. Exploring the psychological responses of people with a high praise-seeking tendency to sincere praise and flattery will help to elucidate the mechanism underlying the socio-emotional effect of praise.

In neuroscience, praise is considered a social reward that activates reward-related brain areas (Izuma et al., 2008; Lin et al., 2012). Reward-related areas respond to material rewards, such as food and money, and the expectation thereof (Schultz, 2000; Knutson et al., 2001; Kirsch et al., 2003; Zald et al., 2004). Systematic reviews have shown that positive rewards activate the nucleus accumbens (NAc), orbitofrontal cortex (OFC), and posterior cingulate cortex (PCC; Liu et al., 2011; Clithero and Rangel, 2013). Several functional magnetic resonance imaging (fMRI) studies have shown that praise and related social rewards, such as a positive impression of subjects by third-party evaluators (Izuma et al., 2008) and a “happy face” accompanied by an emotional sound (Lin et al., 2012), activate many brain areas including the NAc, OFC, and PCC.

However, brain-imaging studies have not examined whether the reliability of praise affects activity in reward-related brain areas. In particular, no such studies have compared sincere praise and flattery. An fMRI study showed that reading positive words led to brain activity in several regions, including the NAc (Hamann and Mao, 2002), implying that reward-related areas can be activated by positive feedback unrelated to performance. Previous studies have also suggested an effect of feedback reliability on brain activity. Electroencephalography studies have shown that unreliable feedback (75% accurate positive feedback) elicits lower amplitudes than reliable feedback (100% accurate positive feedback; Ernst and Steinhauser, 2018). The neural correlates of individual differences in the praise-seeking tendency are not clear. A previous study showed different patterns of neural activation, depending on the reward type and individual differences (Chan et al., 2016), however, the neural basis of the praise-seeking personality is not clear. The individual sensitivity of behavioral inhibition and activation systems in healthy populations has been related to praise-seeking (Kojima, 2018), and was associated with brain activation in the NAc, cingulate, and cuneus in response to a positive face (Radke et al., 2016). Determining the association between the praise-seeking trait and differential neural responses to the two types of praise exploratively may help to elucidate the psychological mechanism underlying the trait, given that it is characterized by differential emotional responses to positive and negative feedback (Kojima et al., 2003).

The purpose of this study was to determine whether feedback reliability affects the rewarding effect of praise, as reflected in the activation of reward-related areas, and to explore the neural correlates of praise-seeking. Participants performed a visual search task under time pressure and received sincere praise or flattery for their performance, distinguished by different face avatars. We expected that sincere praise would elicit more brain activity in reward-related areas than flattery. We also expected that the difference in brain activity would correlate with different perceived reliability. We examined differences in brain activity between the two types of praise, and the correlation between brain activity and the different perceived reliability of the praise associated with each face avatar. In addition, as we anticipated that brain activity would differ depending on the praise-seeking, we explored differences in brain responses to sincere praise and flattery according to the praise-seeking trait, where sincere praise condition constituted negative feedback (i.e., was given after low task performance).

## 2 Method

### 2.1 Subjects

Thirty-four right-handed students from Tohoku University, Japan, participated in the experiment (12 females and 22 males; age range: 18–25 years; mean age = 21.1 ± 1.8 years). Handedness was evaluated using the Edinburgh Handedness Inventory (Oldfield, 1971). Participants had a normal or corrected-to-normal vision and no history of neurological or psychiatric disease. Participants provided informed consent; if they were younger than 20 years, parental consent was also obtained. The study was approved by the Institutional Review Board of the School of Medicine, Tohoku University, Japan (approval no. 2018-1-607) and was conducted according to the principles of the Declaration of Helsinki.

### 2.2 Task and feedbacks

**Figure 1:**
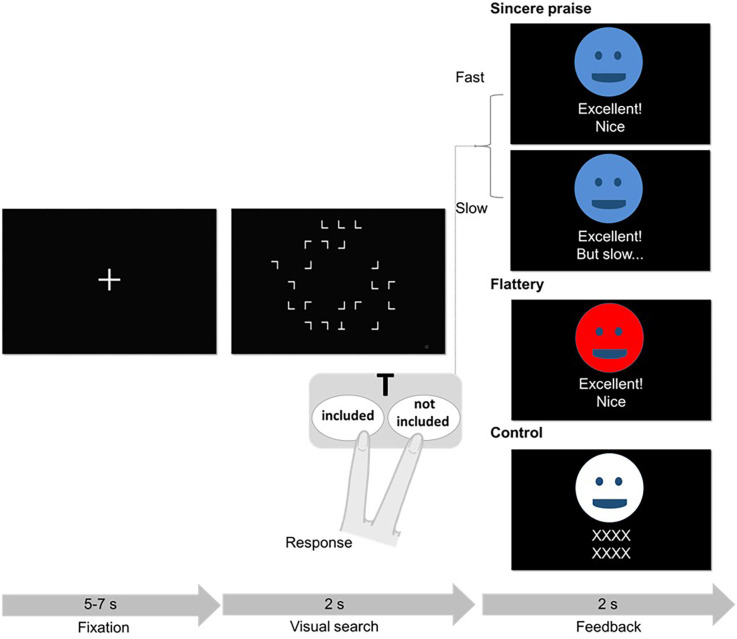
Schematic of the visual search trial and feedback. One of the three face avatars provided feedback following each trial. The association between color (blue, red, or white) and feedback condition (sincere, flattery, or control) was randomized across participants. In the sincere praise condition, different feedback was provided depending on the participant’s reaction time (fast or slow). In the flattery condition, participants always received the same feedback. In the control condition, a string of “X”s was presented. Although not shown here, colored face avatars provided feedback on mistakes in the sincere praise (“Oh my, incorrect”), flattery (“Excellent! Nice”), and control (“XXXX XXXX”) conditions. If a response was not received in time, a face avatar presented the words “Time up (no answer).”

In each trial, participants performed a visual search task and received different types of feedback on their performance: sincere praise, flattery, or meaningless feedback (control; Figure 1). During the visual search task, the letters “L” and “T” were presented in a random orientation and position on an 8 × 6 grid. Each trial used a different stimulus: half of the stimuli were “L”s (*n* = 21), while the other half comprised 20 “L”s and one “T”. Participants were asked to respond as quickly and accurately as possible and provided mostly correct answers.

After each trial of the task, one of the three types of feedback was provided to the participants. In the sincere praise condition, different feedback was provided depending on the participant’s reaction time (e.g., “Excellent! Nice” for fast responses and “Excellent! But slow” for slow responses). To differentiate between the fast and slow conditions, the “standard response time” was set as the cut-off. The standard response time was calculated for each participant, separately for the trials with and without “T”, to ensure an equal number of fast and slow conditions in each trial. The standard response time was the average reaction time in the previous session (e.g., the mean reaction time for session 1 was used as the standard response time for session 2, and the standard response time for session 1 was calculated by reference to the practice session; see Supplementary Data for details). For incorrect answers, “Oh my, incorrect” was displayed. In the flattery condition, participants were always given the same feedback (“Excellent! Nice”) regardless of their performance (fast or slow). However, for incorrect answers, “Oh my, incorrect” was displayed. In the control condition, a string of “X”s was displayed. To ensure that the three types of feedback were clearly distinguishable, each feedback type was presented by a different-colored face avatar. The praise conditions were ordered randomly so that participants could not predict the praise that would follow their response.

### 2.3 Procedure

Before fMRI, participants practiced the visual search task to improve their accuracy and reaction times, and to learn the relationship between the face avatar’s color and praise type. Each participant performed three practice sessions (30 trials in each session; 90 trials in total). Before the practice sessions, participants were informed that the three different face avatars would provide different types of praise/feedback after the visual search task. Participants were instructed to press a button with their right index (or middle) finger when the letter “T” was present and to press the button with their right middle (or index) finger when the letter “T” was not present. The assignment of fingers to each button was counterbalanced across participants. The average reaction time in the practice session was taken as the standard response time for the first MRI experiment (Supplementary Data).

Each participant lay in a supine position on the bed of the MRI scanner, with their heads immobilized. The tasks and feedback were presented on an MRI-compatible LED monitor (BOLD screen 32 LCD for fMRI; Cambridge Research Systems, Rochester, UK). Each participant viewed the monitor *via* a mirror attached to the head coil, with the LCD display placed behind the head coil. While inside the scanner, each participant performed three task sessions (48 trials each; 144 trials in total). Participants were instructed to perform the task using the keys on an MRI-compatible response box held in the right hand, with the same fingers used in the practice session.

### 2.4 fMRI data acquisition

The fMRI scans were performed using a Philips Achieva 3 Tesla scanner (Philips Healthcare, Best, The Netherlands) with an eight-channel head coil. Because our regions of interest (ROIs) were near brain areas with relatively high magnetic inhomogeneity, we used a dual-echo sequence for data acquisition (Schwarzbauer and Porter, 2010; Halai et al., 2014), which is resistant to magnetic-susceptibility artifacts. The continuous dual gradient-echo sequence included 38 slices that covered the entire brain, with short and long echo times (TEs) of 13 and 35 ms, respectively, a repetition time (TR) of 2,000 ms, 64 × 64 acquisition matrix, field of view (FOV) of 240 mm, in-plane resolution of 3.75 × 3.75 mm, and slice thickness of 4 mm (without a gap). Each session lasted for 530 s (265 volumes; 795 volumes in total, i.e., three sessions, acquired in 26 min 30 s). A high-resolution T1-weighted structural image was acquired for spatial normalization using an MP-RAGE sequence (TR = 6.6 ms, TE = 3 ms, matrix size = 240 × 240, FOV = 240 mm, number of slices = 162, slice thickness = 1 mm).

### 2.5 Task impression questionnaire

Participants completed a questionnaire comprising four items on perceptions of the praise/feedback and face avatar in the three conditions (scored from 1 to 8; 1: strongly disagree; 8: strongly agree), before (i.e., after the practice session) and after the fMRI task; we used the average score for the analysis to reflect impressions throughout the fMRI task.

Feedback perception was measured by the following two items: “The feedback from the (blue/red/white) face avatar did not depend on your performance” (Q1: perceived reliability) and “You felt flattered when the (blue/red/white) face avatar gave you feedback” (Q2: perceived flattery). To calculate the perceived reliability of the sincere praise compared to flattery, we determined the difference in Q1 (perceived reliability) scores (reverse-scored) between the sincere praise and flattery conditions (i.e., sincere praise score − flattery score); this difference was called the reliability score. Q2 (perceived flattery) was used to determine whether participants perceived the flattery condition to be akin to “flattery in daily life”.

The socio-emotional effects of praise were assessed by the following two statements pertaining to the praising face avatar: “You were pleased to receive feedback from the (blue/red/white) face avatar” (Q3: feeling of happiness) and “You liked the (blue/red/white) face avatar” (Q4: preference for face avatar). We calculated the difference in scores between the sincere praise and flattery conditions (i.e., sincere praise score − flattery score), which confirmed a difference between the conditions. In addition, we found positive correlations between the questionnaire scores and praise-seeking traits.

To compare the scores for Q1–Q4 among the three praise types, we conducted a one-way within-subjects analysis of variance (ANOVA) using an online tool (ANOVA4^1^). *Post-hoc* corrections for multiple comparisons were conducted using Ryan’s method. In addition, we examined the correlation of the praise-seeking trait with the scores for Q3 (feeling of happiness) and Q4 (preference for face avatar), which were likely to be influenced by praise-seeking, using Pearson’s correlation.

### 2.6 Personality trait questionnaires

Before fMRI, participants completed a personality questionnaire to measure their tendency for praise-seeking (Kojima et al., 2003). To explore the possibility that the relationships between neural activation sites and praise-seeking may be explained by other personality traits, 15 indices from five questionnaires were used (Supplementary Data).

### 2.7 fMRI data pre-processing

Pre-processing and statistical analysis were performed using Statistical Parametric Mapping (SPM8) software (Wellcome Centre for Neuroimaging, London, UK). Those whose head movements during scanning that exceeded the acquired voxel size (>3.75 mm) were excluded from the analysis. After correcting for head motion using the standard realignment procedure of SPM8, short- and long-echo images were linearly combined with equal weighting (Schwarzbauer and Porter, 2010; Halai et al., 2014). The pre-processing procedure also included slice-timing correction, spatial normalization using a T1-based deformation field with resampling to an isotropic 2-mm voxel size, and spatial smoothing using a Gaussian kernel of 8-mm full width at half maximum.

### 2.8 Within-subject analysis of fMRI data

For within-subject analysis, multiple regression analysis of the expected signal change over time was performed, on a voxel-by-voxel basis, on pre-processed images using visual search task (duration: 2 s), response (0 s), and feedback (2 s) as the regressors in the general linear model. Feedback was grouped by praise condition and task performance: sincere praise-fast, sincere praise-slow, flattery-fast, flattery-slow, control-fast, and control-slow. When a trial could be not categorized due to aberrant response speed (i.e., all fast or all slow responses in any condition), the corresponding regressor was omitted and the contrast vector was modulated so that the sum of the weights for any comparison was 0 (e.g., if one of the sessions lacked the sincere praise-slow condition, the weight for the same condition in the other two sessions would be set to 1.5 instead of 1, to balance it with the control condition having three regressors with a weight of −1). Cases with an incorrect or no response were labeled “condition of no interest” (*N* = 3). The six realignment parameters of the estimated head movement were “covariates of no interest”. A high-pass filter with a cut-off frequency of 1/128 Hz was used for detrending.

### 2.9 Between-subject analysis of fMRI data

For the between-subject analysis, depending on the hypothesis, contrast images were created to compare each condition with the control condition, without or with consideration of speed (speed-non-distinctive and speed-distinctive contrast, respectively) relative to the control condition (fast or slow). Speed-non-distinctive contrasts were created by pooling the speed-distinctive contrasts. Speed-non-distinctive contrasts were used to examine the effect of feedback type independent of performance (performance-independent), whereas speed-distinctive contrasts were used to examine the performance-dependent effect of feedback type. After estimating the contrast maps, we conducted group-level statistical inference focusing on reward-related areas (i.e., ROI analysis), followed by whole-brain analysis.

**Figure 2:**
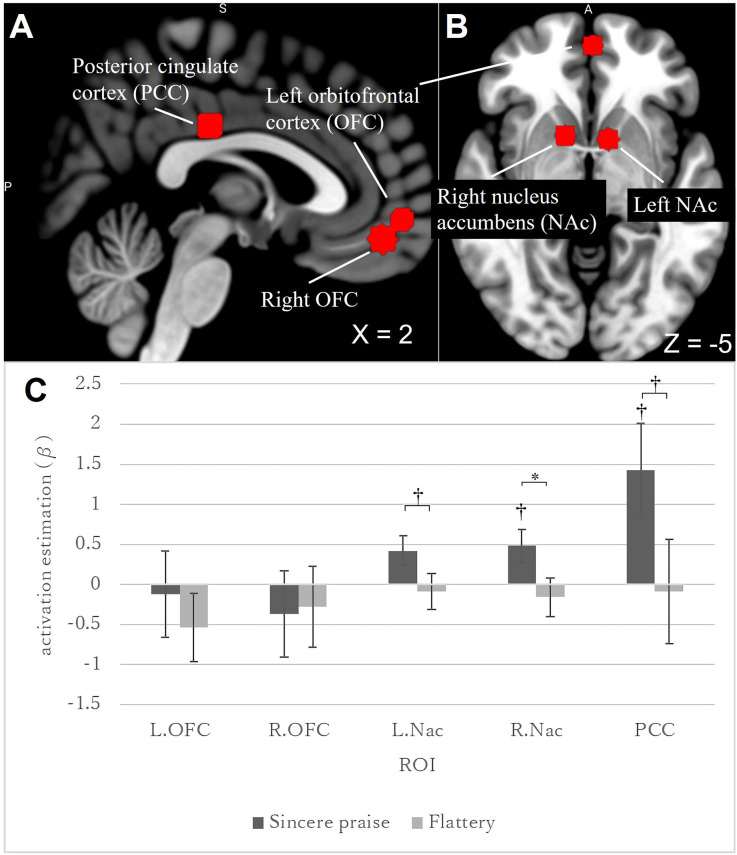
Regions of interest (ROIs), and brain activities in these ROIs in response to feedback (sincere praise and flattery), compared to the control. A brain slice at the following MNI coordinates: **(A)** *x* = 2; **(B)** *z* = −5. ROIs are marked with a red sphere [**(A)** PCC and bilateral OFC; **(B)** bilateral NAc and left OFC], and were produced using Wake Forest University PickAtlas software. Brains were mapped onto the MNI template brain using MRIcroGL (https://www.mccauslandcenter.sc.edu/mricrogl/home). PCC, posterior cingulate cortex; OFC, orbitofrontal cortex; NAc, nucleus accumbens. Bar graph **(C)** showing the brain activity elicited by sincere praise and flattery in ROIs (bilateral OFC, bilateral NAc, and PCC) with no speed-distinctive contrast [*: Bonferroni-corrected *p*-value (0.05/5); ^†^: uncorrected *p*-value < 0.05]. In the right NAc, brain activity was significantly greater during sincere praise than flattery [Bonferroni-corrected *p*-value (0.016/5)]. NAc, nucleus accumbens; OFC, orbitofrontal cortices; PCC, posterior cingulate cortex.

Five ROIs were drawn for reward-related areas: the right NAc [Montreal Neurological Institute (MNI) coordinates: +12, +10, −6], the left NAc (−10, +8, −4), the right OFC (+2, +48, −14), the left OFC (−2, +56, −6), and the PCC (0, −22, 32). The MNI coordinates were based on a meta-analysis by Liu et al. (2011); (Figures 2A,B). Six-millimeter spherical ROI masks were produced using Wake Forest University PickAtlas software (Maldjian et al., 2003). The average voxel value in each ROI was calculated. A *p*-value < 0.05 was considered significant. The Bonferroni correction (0.05/5; the threshold was corrected by the number of ROIs) was used for multiple comparisons of the five ROIs. We did not use anatomical ROIs given the spatial mismatches between functional and anatomical images attributable to susceptibility artifacts (see Supplementary Methods for details).

For the voxel-wise whole-brain analysis, conducted using SPM8, *p* < 0.001 was used as the cluster-defining threshold [derived from *p* < 0.05 based on family-wise error (FWE)]. This correction was made for all following whole brain analyses. Anatomical labeling of activated areas was based on the Automated Anatomical Labeling Atlas (Tzourio-Mazoyer et al., 2002).

#### 2.9.1 Sincere praise vs. flattery

To compare the performance-independent effects of sincere praise and flattery, differences in brain activation between sincere praise and flattery were examined by one-sample *t*-tests in 5 ROIs and by whole-brain analysis, using the speed-non-distinctive contrast. The brain activations with feedback (sincere praise and flattery) were also assessed by comparison with the control condition. We also compared the neural activation associated with sincere praise and flattery between fast and slow responses by one-sample *t*-tests in 5 ROIs and by whole-brain analysis, using the speed-distinctive contrasts (fast and slow).

#### 2.9.2 Correlation with reliability

The association of reliability scores (sincere praise − flattery) and differential brain activation was examined using the speed-non-distinctive contrast. Pearson’s correlation was used for the ROI analysis. Whole-brain voxel-by-voxel regression analysis was performed based on the reliability score. For reference purposes, the association between brain activation and reliability in the fast and slow conditions was examined using the speed-distinctive contrasts (fast and slow).

#### 2.9.3 Correlation with praise-seeking

Sincere praise condition and flattery condition for low performance are negative and positive feedbacks, respectively. To explore the neural correlates of praise-seeking, correlation analyses were performed between praise-seeking and reliability scores (sincere praise − flattery) in the slow condition using speed-distinctive contrasts. Pearson’s correlation was used for the ROI analysis. Whole-brain voxel-by-voxel regression analysis was performed based on the praise-seeking score. For reference purposes, the overall association between brain activation and praise-seeking, and that in the fast condition, was also examined using speed-non-distinctive and -distinctive contrasts (fast).

For brain regions found to be associated with praise-seeking, we examined their associations with related personality traits. Because 15 personality indices were considered, Bonferroni correction was used for multiple comparisons.

## 3 Result

### 3.1 Behavioral data

#### 3.1.1 Data selection

Two of the 34 participants were excluded from the analysis due to low accuracy or speed of responses in some of the visual search task conditions, i.e., <50% of trials completed correctly or in time. One participant was excluded due to head movement during scanning that exceeded the acquired voxel size (>3.75 mm). In total, data from 31 participants were analyzed (11 females and 20 males; mean age: 21.2 ± 1.8 years). There is no established power analysis methods for fMRI because the statistical power depends not only sample size but also experimental design (Mumford, 2012). We exceeded the number of samples that the previous study showed as necessary (Desmond and Glover, 2002).

#### 3.1.2 Accuracy and reaction time

We conducted a one-way within-subjects ANOVA of reaction time and accuracy in the visual search task. The mean accuracy rates for the sincere praise, flattery, and control conditions were 85.8 ± 9.4%, 87.0 ± 7.4%, and 85.3 ± 8.8%, respectively. The mean reaction times for the sincere praise, flattery, and control conditions were 1.17 ± 0.16 s, 1.15 ± 0.13 s, and 1.14 ± 0.17 s, respectively. There were no significant differences in accuracy (*F*_[2,60]_ = 0.789, *p* = 0.459) or reaction time (*F*_[2, 60]_ = 0.905, *p* = 0.410) between the praise types.

#### 3.1.3 Feedback perception

The mean Q1 (perceived reliability) score was higher for sincere praise than flattery and control conditions, and for flattery than the control condition. The mean Q2 (perceived flattery) score was higher for the flattery than sincere praise and control conditions, with no significant difference seen between the sincere praise and control conditions (Supplementary Data).

#### 3.1.4 Impression of praising face avatar

The mean Q3 (feeling of happiness) score was higher for the sincere praise than flattery and control conditions, and for the flattery than the control condition. The score difference between the sincere praise and flattery conditions for Q3 (feeling of happiness) did not correlate with the praise-seeking score. The mean Q4 (preference for face avatar) score was higher for the sincere praise than flattery and control conditions, and for the flattery than control condition. The score difference between the sincere praise and flattery conditions for Q4 (preference for face avatar) significantly correlated with the praise-seeking score (*r* = −0.420, *t* = 2.496, *p* = 0.019; Supplementary Data).

### 3.2 fMRI data

#### 3.2.1 Sincere praise vs. flattery

Among the five ROIs, a significantly stronger response was identified in the right NAc [sincere praise > flattery, *p*-corrected = 0.0039 × 5 (the threshold was corrected for the number of ROIs following Bonferroni’s method)] to sincere praise compared to flattery (Figure 2C). There was no significant difference in brain activity between the sincere praise and flattery conditions and the control condition. These results were replicated at a liberal statistical threshold when speed was accounted for (Supplementary Figure 1).

**Figure 3:**
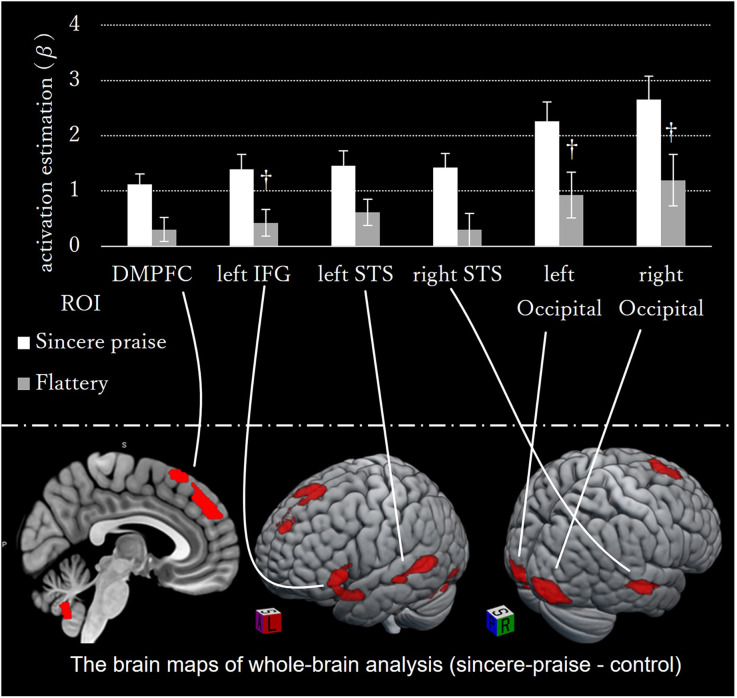
Whole-brain activation during sincere praise compared to the control condition (speed-non-distinctive contrast). The brain maps on the bottom panel of whole-brain analysis comparing the sincere praise and control conditions (speed-non-distinctive contrast). Bar graph of the brain activity in ROIs in the sincere praise and flattery compared to the control conditions (^†^: Bonferroni-uncorrected *p*-value < 0.05). Activation of the dorsal medial prefrontal cortex (DMPFC), left inferior frontal gyrus, and right superior temporal sulcus occurred only in response to sincere praise. Activity in DMPFC, left IFG, left STS, and right STS in the Flattery condition is insignificant in response to flattery. Brains were mapped onto the MNI template brain using MRIcroGL (https://www.mccauslandcenter.sc.edu/mricrogl/home).

No significant regions of contrast were noted between the sincere praise and flattery conditions in the whole-brain analysis. Comparison of the sincere praise and control conditions revealed significantly greater activation of the dorsal medial prefrontal cortex (DMPFC), left inferior frontal gyrus (IFG), bilateral superior temporal sulcus (STS), and occipital lobe in the former condition (Figure 3, Table 1). To confirm that this brain activity was unique to sincere praise and not found in flattery, we also examined the brain activity in these regions in the flattery condition, and compared it to the control condition. Note that, because the activity in this region differed between sincere praise and control, there is a concern for biased deactivation in the control condition, yielding a false-positive on the comparison of flattery and control. Activation of the DMPFC, right STS, and left IFG was seen in response to sincere praise but was not significantly different between the flattery and control conditions at a lenient threshold (*p* > 0.05, uncorrected following Bonferroni’s method).

**Table 1:** Differences in brain activation by praise type.

Comparison	Location	Side	MNI coordinates			Voxel size (k)	Cluster *p*-value (FEW-corrected)	FWHM (mm)			Resolution element (resels)
			x	y	z			x	y	z	
Sincere praise − Control	Lingual	L	–32	−92	−14	1,952	<0.001	16.5	16.6	12.9	397.9
			−18	−94	−20						
			−46	−54	−24						
	Inferior occipital	R	26	−92	−10	616	0.004				
	Middle temporal	L	−60	−52	6	604	0.004				
			−50	−42	0						
			−62	−24	−6						
	Superior medial frontal	R	4	24	60	821	0.001				
			6	44	42						
			0	50	30						
	Middle temporal	R	58	−24	−6	621	0.003				
	Superior temporal pole	L	−46	24	−16	533	0.007				
			−50	24	0						
			−54	10	−18						
Flattery − Control	n.s.							17.6	17.5	13.4	338.9
Sincere praise − Flattery	n.s							17.5	17.6	13.6	337.2

Table 1 shows the results of whole-brain analysis of activated brain areas without consideration of speed (sincere praise and flattery conditions compared to the control; FEW cluster *p*-value < 0.05). For each region, the location and coordinates of the activation peak in MNI space, activated cluster size in number (*k*) of voxels (2 × 2 × 2 mm^3^), *p*-value, and the size of the resolution element (i.e., representing spatial autocorrelation) for the correction for multiple comparisons (FWHM and RESEL count) are listed. To identify the activated areas, the Anatomical Automatic Labeling (AAL) atlas was used. FEW, family-wise error; n.s., not significant; L, left; R, right; FWHM, Full Width at Half Maximum; RESEL, Resolution Element.

In the whole-brain analysis, there were no significant regions of contrast between the flattery and control conditions. The areas differentially activated between conditions according to speed are listed in Supplementary Table 1.

#### 3.2.2 Correlation with reliability

**Figure 4:**
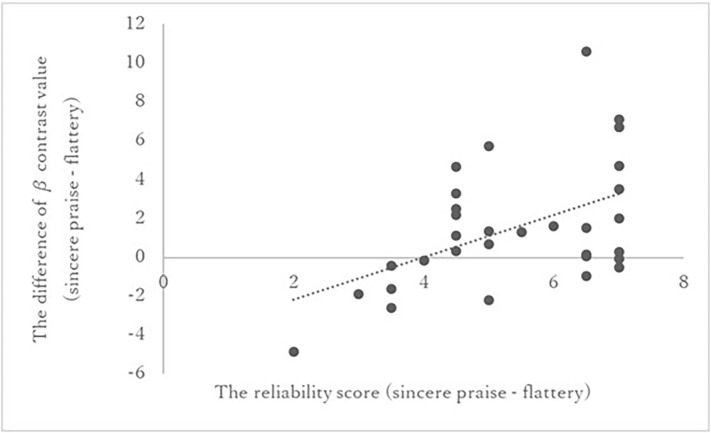
Correlation between reliability score and PCC activation in the sincere praise and flattery conditions. Pearson’s correlation between the reliability score and activity in the PCC. Higher reliability scores correlated with greater activity in the PCC during sincere praise compared to flattery. PCC, posterior cingulate cortex.

Among the five ROIs, a significant correlation was identified between PCC activity and reliability [*r* = 0.491, p-corrected = 0.01 × 5 (the threshold was corrected for the number of ROIs following Bonferroni’s method); Figure 4]. No significant correlations were found between the other ROIs and speed (left OFC: *r* = 0.03, right OFC: *r* = 0.02, left NAc: *r* = 0.22, right NAc: *r* = 0.24). The correlations of the ROIs with speed (overall and fast) are listed in Supplementary Table 2. Whole-brain regression analysis of the reliability score did not produce any significant results.

#### 3.2.3 Correlation with praise-seeking

No significant correlations were identified between the activity in the five ROIs and praise-seeking (left OFC: *r* = 0.067, right OFC: *r* = 0.038, left NAc: *r* = −0.104, right NAc: *r* = −0.358, PCC: *r* = 0.059). The correlations of the ROIs with speed (overall and fast) are listed in Supplementary Table 3.

**Figure 5:**
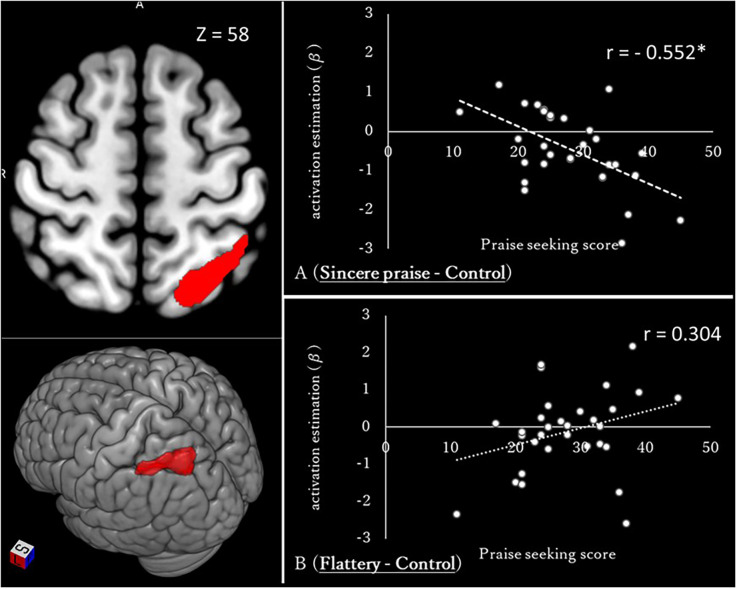
Whole-brain regression analysis of praise-seeking (sincere praise − flattery) and correlation between brain activation and the praise-seeking score [(sincere praise − control) and (flattery − control)] The images on the left show the intraparietal sulcus, the activity in which was identified using whole-brain regression analysis according to praise-seeking and speed-distinctive (slow) contrasts [cluster-defining threshold: uncorrected *p*-value < 0.001, derived from *p* < 0.05 based on *via* family-wise error (FWE)]. Brains were mapped onto the MNI template brain using MRIcroGL (https://www.mccauslandcenter.sc.edu/mricrogl/home). Diagrams on the right show the correlation between brain activation and the praise-seeking trait (*p < 0.05).

The whole-brain analysis revealed a significant negative correlation between left intraparietal sulcus (IPS) activation and praise-seeking (Figure 5, Table 2). To determine whether this correlation pertained to sincere praise or flattery, and involved activation or deactivation, a comparison of average values among subjects (one-sample *t*-test) and correlation analysis between the praise-seeking score and brain activity during the sincere praise and flattery conditions (compared to the control) were conducted. The sincere praise condition was associated with significantly less brain activity than the control condition (*β* = −0.449, *p* = 0.001), but there was no significant association in the flattery condition (*β* = −0.116, *p* = 0.555). Relative to the control condition, there was a significant negative correlation between the sincere praise condition and praise-seeking (*r* = −0.552, *p* = 0.001), but no significant correlation for the flattery condition (*r* = 0.304, *p* = 0.096).

**Table 2:** Brain regions associated with praise-seeking tendency.

Comparison	Speed	Location	Side	MNI coordinate			Voxel size	Cluster *p*-value	FWHM (mm)			Resolution element (resels)
				x	y	z	(k)	(FWE-corrected)	x	y	z	
Flattery—Sincere praise	Slow	Superior parietal	L	−34	−62	58	527	0.004	16.0	15.9	12.3	450.6
				−46	−48	58						
				−28	−72	54						

Table 2 shows the region identified by whole-brain regression analysis as being associated with the praise-seeking tendency (FWE cluster *p*-value < 0.05). For each region, the location and coordinates of the activation peak in MNI space, activated cluster size in number (*k*) of voxels (2 × 2 × 2 mm^3^), *p*-value, and the size of the resolution element (i.e., representing spatial autocorrelation) for the correction for multiple comparisons (FWHM and RESEL count) are listed. To identify the activated locations, the Anatomical Automatic Labeling (AAL) atlas was used. FWE, family-wise error.

To rule out the possibility that the correlation between left IPS activation and the praise-seeking score may have been due to other personal characteristics, correlations between brain activity in the left IPS and the 15 personality indices were examined (Supplementary Table 4). A modest correlation was found between the BAS-FS (fun-seeking) and left IPS activation.

## 4 Discussion

The aims of the present study were to investigate the rewarding effect of the reliability of praise and explore the cognitive processes responsible for individual differences in the praise-seeking trait. We identified significantly greater brain activity in the right NAc in the sincere praise compared to the flattery condition; participants with higher reliability scores for praise exhibited greater activity in the PCC in the sincere praise compared to flattery condition. We also identified a negative correlation between praise-seeking and the difference in activation of the left IPS (sincere praise − flattery conditions); the IPS was deactivated in the sincere praise condition in high praise-seekers.

### 4.1 Rewarding effect

Our results support a rewarding effect of reliable praise. NAc activation is considered to reflect a rewarding effect, where the NAc shows dopaminergic innervation (Izuma et al., 2008; Lin et al., 2012); sincere praise (i.e., reliable praise) evokes more brain activity in the NAc compared to flattery, suggesting a higher rewarding effect of the former. The PCC, rather than dopamine-innervated areas, is involved in higher-order processing of social interactions (Yokoyama et al., 2017; Alcalá-López et al., 2018; Kageyama et al., 2019), probably based on self-referential processing (Brewer et al., 2013), regulating the focus of attention (Leech and Sharp, 2014), and in the functional processing and flexible response to environment changes (Leech et al., 2012). The correlation between the brain activity in the PCC and subjective reliability scores of our participants may reflect the high cognitive process, engagement with information relevant to the self, and adaptation of oneself to the environment, mirroring the rewarding effects of reliable feedback.

Reliable sincere praise may have a rewarding effect because reliability promotes accurate social perceptions. Human communication can contain misunderstanding or even deliberate lies (Koenig et al., 2004); therefore, judicious use of social information is important for survival (Laland, 2004). In addition, an accurate understanding of others’ intentions has social survival value. The whole-brain comparison between the sincere praise and control conditions in this study supports the aforementioned view, where there were differences in activation of the DMPFC, left IFG, and right STS. The DMPFC is involved in performance monitoring and modulates reward value (Duverne and Koechlin, 2017), while the left IFG and superior temporal region are involved in semantic processing (Rodd et al., 2005; Binder et al., 2009). Activation of these brain areas may reflect the understanding of the reliability of feedback, performance monitoring, and behavior modification.

### 4.2 Socio-emotional effect

The neural correlates of praise-seeking suggest the involvement of attentional processes. People with a high praise-seeking tendency appreciate praise and attribute negative feedback to someone or something other than themselves (Kojima et al., 2003). This biased perception was reflected in the answers to Q4 (preference for face avatar) in this study, where the participants preferred the flattering avatar over sincere praise avatar, which may be due to attentional suppression. Analysis of the neuroimaging data for the slow condition showed a stronger negative correlation between praise-seeking and brain activity in the left IPS in the sincere praise compared to the flattery condition, due to deactivation of the left IPS in high praise-seeking individuals. The IPS is part of the dorsal attentional system and is involved in top-down attentional control (Vossel et al., 2014). The perceptual bias associated with the praise-seeking trait may reflect IPS deactivation.

This top-down attentional control may be “generalized” due to positive illusion, which involves biased perceptions that maintain self-esteem. It has long been thought that positive illusions are necessary for good mental health; they also aid adaptation (Taylor and Brown, 1988; Shedler et al., 1993). Previous studies revealed that people with a high praise-seeking tendency are more likely to view past events in a positive way (Kishida et al., 2016), which amounts to a positive illusion. The mechanism for suppressing negative feedback can in fact be applied to positive illusion, as can the mechanism underlying praise-seeking (i.e., top-down attentional control) found in this study.

### 4.3 Different effects of praise

In this study, we revealed the neural dynamics of the different effects of praise (i.e., rewarding and socio-emotional). The rewarding effect, reflected by the activation of reward-related areas, depends on the reliability of the praise (i.e., performance feedback), and is higher for sincere praise than flattery. In contrast, the socio-emotional effect is based on the positive feedback conveyed by the praise, and is enhanced by filtering out negative feedback in individuals who have a high praise-seeking tendency. Since these two effects sometimes conflict with each other, individual differences in the value of the latter effect have caused controversy regarding the value of flattery, especially in marketing studies (Main et al., 2007; Chan and Sengupta, 2010).

### 4.4 Tailor-made conversations

These results may be useful for communication studies, in particular, those on “tailor-made conversations”. With the development of artificial intelligence, studies on appropriate communication based on this technology are gaining importance. In the present experiment, we found that the effectiveness of the communication was affected by praise reliability and the personality of the individual being praised. Advanced techniques to estimate the praise-seeking tendency of individuals may be useful to identify the most appropriate words for praising individuals differing in personality and background.

### 4.5 Limitation

A methodological limitation of the present study was the definitions of two types of praise. Our definitions are based on the previous definitions of sincere praise as performance-dependent praise and flattery as performance-independent; thus, the effects of relationship context and the situation could not be considered. However, flattery may have a social motive behind it; it may be possible to define flattery only when the actual performance is not worthy of praise. It should be noted that compared to flattery defined in a richer and more ecological social context, our “flattery” lacks such comprehensive characteristics, and the findings of our study are thus somewhat limited. Future analyses of praise should consider these factors.

## Data availability statement

The raw data supporting the conclusions of this article will be made available by the authors, without undue reservation.

## Ethics statement

The studies involving human participants were reviewed and approved by The Institutional Review Board of the School of Medicine, Tohoku University, Japan (approval no. 2018-1-607). The patients/participants provided their written informed consent to participate in this study.

## Author contributions

SF: conceptualization, methodology, formal analysis, investigation, writing—original draft, and visualization. RI: conceptualization, methodology, software programming, validation verification, formal analysis, investigation, data curation, writing—review and editing. AT-I: methodology, software programming, investigation, writing—review and editing. RK: resources, writing—review and editing, funding acquisition. MS: resources, writing—review and editing, visualization, supervision, project administration, and funding acquisition. All authors contributed to the article and approved the submitted version.

## Footnotes

^1^ https://www.hju.ac.jp/ kiriki/anova4/
