# Supplementary material for: Sincere praise and flattery: reward value and association with the praise-seeking trait
Source: Front Hum Neurosci. 2023 Feb 15;17:985047. doi: 10.3389/fnhum.2023.985047 (PMC9974641; doi:10.3389/fnhum.2023.985047)
Supplement: Supplementary file 1 [file Data_Sheet_1.docx]

Supplementary Material

# Supplementary Methods

## Standard response time

The standard response time for the initial fMRI was determined based on the practice sessions. In the first practice session, the standard response time for the visual search task including “T” as a stimulus was set to 1.0 s, while the standard response time for the task without “T” was set to 1.5 s. The average reaction time in the first session was set as the standard reaction time for the second session, and the average reaction time in the second session was set as the standard reaction time for third session. The standard response time for the first MRI experiment was set based on the average reaction time in the third practice session.

## Questionnaires

We also administered the Self-Esteem Scale (Rosenberg, 1965; Yamamoto et al., 1982), Big-Five personality inventory (Gosling et al., 2003; Oshio et al., 2012), BIS/BAS (behavioural approach system/ behavioural inhibition system)Scales (Carver and White, 1994; Takahashi et al., 2007), Effortful Control Scale (Rothbart et al., 2000; Yamagata et al., 2005), and Daily Life Skills Scale (Shimamoto and Motonobu, 2006).

## The ROIs

As the NAc is not an anatomically large, it may be better to recruit an anatomical NAc when examining brain activity. However, we constructed sphere ROIs with a radius of 6 mm centered at the coordinates of the NAc, as defined by a meta-analysis of fMRI studies using Echo Planer Imaging (EPI) by Liu et al. 2011 (n=142 studies); we did not use anatomical ROIs. Given the basal cerebral void, a large susceptibility artifact near the NAc is unavoidable, and the NAc defined by T1- and T2-weighted anatomical images and the reward-related brain response measured by EPI may not spatially match (Supplementary Figure 2). As we were analyzing EPI images, we thought it appropriate to use the NAc ROI, defined by the reward-related brain response measured via EPI.

# Supplementary Results

## Analysis of Q1 (perceived reliability) scores

The mean Q1 (perceived reliability) scores in the sincere-praise, flattery, and control conditions were 2.66 ± 1.19, 6.53 ± 1.26, and 2.18 ± 1.52, respectively. There was a significant effect of condition in the one-way ANOVA (F[2,60] = 94.276, p < 0.001). The post-hoc paired t-test revealed that the score was higher for the sincere-praise than flattery and control conditions (t = 12.967, p < 0.001 and t = 10.397, p < 0.001, respectively), and for the flattery than control condition (t = 2.570, p = 0.013).

## Analysis of Q2 (perceived flattery) scores

The mean Q2 (perceived flattery) scores in the sincere-praise, flattery, and control conditions were 7.29 ± 0.74, 1.92 ± 1.09, and 2.98 ± 2.42, respectively. There was a significant effect of condition in the one-way ANOVA (F[2,60] = 176.613, p < 0.001). The post-hoc paired t-test revealed that the score was higher for the flattery than sincere-praise and control conditions (t = 10.924, p < 0.001 and t = 12.290, p < 0.001, respectively). However, there was no significant difference between the sincere-praise and control conditions (t = 1.366, p =0.177).

## Analysis of Q3 (feeling of happiness) scores

The mean Q3 (feeling of happiness) scores in the sincere-praise, flattery, and control conditions were 6.24 ± 0.96, 4.53 ± 1.45, and 1.65 ± 0.75, respectively. There was a significant effect of condition in the one-way ANOVA (F[2,60] = 135.103, p<0.001). The post-hoc paired t-test revealed that the score was higher for the sincere-praise than flattery and control conditions (t = 6.048, p < 0.001; t = 16.261, p < 0.001, respectively), and for the flattery than control condition (t = 10.213, p < 0.001). The correlation between the praise-seeking score and difference in Q3 (feeling of happiness) score between the sincere-praise and flattery conditions was not significant (r = −0.295, t = 1.664, p = 0.107). The correlations of the praise-seeking score with the Q3 (feeling of happiness) score differences were as follows: (sincere praise – control: r = 0.105, p = 0.573 and Q3 (feeling of happiness) and flattery – control: r = 0.389, p = 0.030).

## Analysis of Q4 (preference for each face avatar) scores

The mean Q4 (preference for each face avatar) scores in the sincere-praise, flattery, and control conditions were 5.76 ± 1.02, 4.56 ± 1.34, and 2.65 ± 0.94, respectively. There was a significant effect of condition in the one-way ANOVA (F[2,60] = 65.064, p < 0.001). The post-hoc paired t-test revealed that the score was higher for the sincere-praise than flattery and control conditions (t = 4.211, p < 0.001; t = 11.287, p < 0.001, respectively), and for the flattery than control condition (t = 7.076, p < 0.001). The correlation between the praise-seeking score and score difference between sincere praise and flattery for Q4 (preference for each face avatar) was significant (r = −0.420, t = 2.496, p = 0.019). The correlations of the praise-seeking score with the Q4 (preference for each face avatar) score differences were as follows: (Q4 (preference for each face avatar) and sincere praise – control: r = −0.058, p = 0.756 and Q4 (preference for each face avatar) and flattery – control: r = 0.420, p = 0.019).

# Supplementary Figures and Tables

**
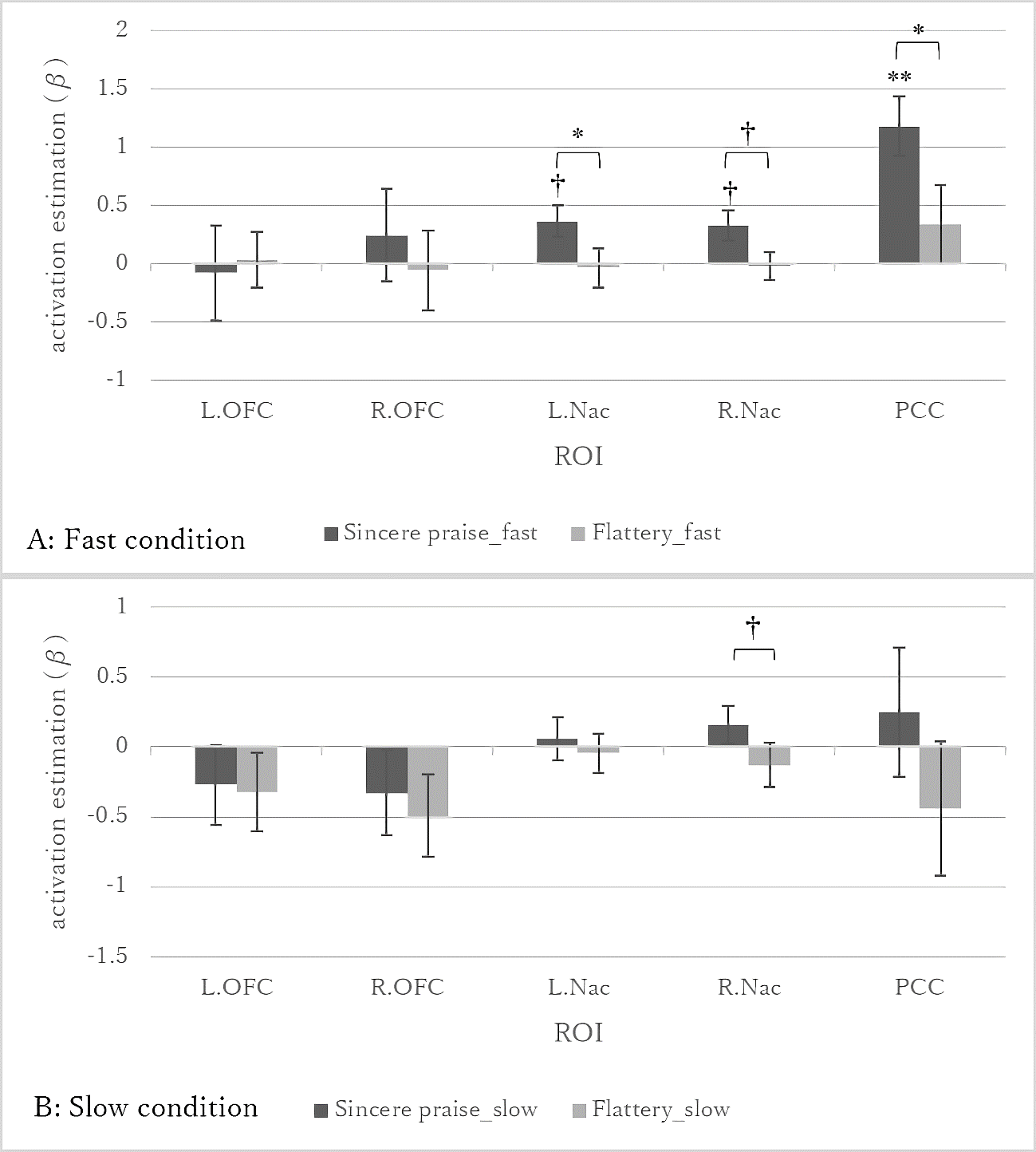
**

**Supplementary Figure 1. Activation in reward-related areas by feedback (sincere praise and flattery compared to control)**

A: fast trials, B: slow trials. NAc: nucleus accumbens, OFC: orbitofrontal cortices, PCC: posterior cingulate cortex. (**: Bonferroni-corrected p-value < 0.01; n = 5; *: corrected p-value < 0.05; †: uncorrected p-value < 0.05).

In the fast condition (Supplementary Figure 1-A), the only factor that was different between the sincere-praise and flattery conditions was reliability, which explained the difference in brain activity between these conditions. In the slow condition (Supplementary Figure 1-B), feedback words differed between conditions. In the sincere-praise condition, the feedback was reliable but did not include praise (“Excellent but slow”). In the flattery condition, the feedback included praise (“Excellent! Nice”) but was not reliable. Under these conditions, praise may lead to both motivational and socio-emotional effects.


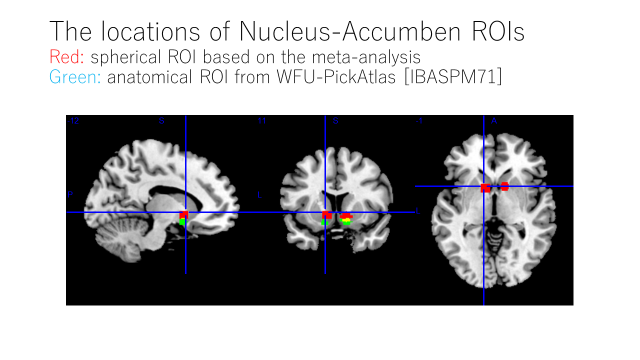


Supplementary Figure 2. ROIs based on the fMRI meta-analysis and the anatomical ROIs

Each red area is a sphere of radius 6 mm, centered at the co-ordinates of the NAc defined in our original ROI (i.e., the meta-analysis (n=142 studies) of the fMRI study using EPI of Liu et al., 2011). The green area is the NAc region defined anatomically in the WFU_pickatlas.

Supplementary Table 1 Activated areas in whole-brain analysis by praise type and speed

| Comparison | Speed | Location | Side | MNI coordinates | | | Voxel size (k) | Cluster p-value (FWE-corrected) |
| --- | --- | --- | --- | --- | --- | --- | --- | --- |
|  |  |  |  | x | y | z |  |  |
| Sincere praise - Control | fast | Superior  medial frontal | R | 4 | 44 | 38 | 1140 | < 0.001 |
|  |  |  |  | 10 | 50 | 38 |  |  |
|  |  |  |  | 2 | 48 | 28 |  |  |
|  |  | Inferior occipital | L | –34 | –88 | –16 | 975 | < 0.001 |
|  |  |  |  | -26 | -98 | -6 |  |  |
|  |  |  |  | -16 | -86 | -22 |  |  |
|  |  | Inferior parietal | R | 46 | –56 | 52 | 483 | 0.012 |
|  |  |  |  | 52 | -52 | 48 |  |  |
|  |  |  |  | 32 | -56 | 40 |  |  |
| Sincere praise - Control | slow | Inferior Occipital | L | –30 | –92 | –12 | 739 | < 0.001 |
|  |  |  |  | -16 | -96 | -18 |  |  |
|  |  |  |  | -44 | -54 | -22 |  |  |
|  |  | Lingual | R | 26 | –92 | –10 | 420 | 0.015 |
|  |  | Middle temporal | L | –60 | –52 | 4 | 507 | 0.007 |
|  |  |  |  | -64 | -32 | -4 |  |  |
|  |  |  |  | -60 | -22 | -6 |  |  |
|  |  | Middle temporal | R | 58 | –24 | –4 | 386 | 0.021 |
| Flattery - control | fast | n.s. |  |  |  |  |  |  |
| Flattery - control | slow | n.s. |  |  |  |  |  |  |
| Sincere praise - flattery | fast | n.s. |  |  |  |  |  |  |
| Sincere praise - flattery | slow | n.s. |  |  |  |  |  |  |
| Flattery - sincere praise | fast | n.s. |  |  |  |  |  |  |
| Flattery - sincere praise | slow | Postcentral | R | 38 | –36 | 48 | 384 | 0.018 |
|  |  |  |  | 34 | -36 | 48 |  |  |
|  |  |  |  | 24 | -46 | 58 |  |  |

Supplementary Table 1 shows the results of whole-brain analysis of activated areas according to speed (FWE cluster p-value < 0.05). For each region, the location and coordinates of the activation peak in MNI space, activated cluster size in number (k) of voxels (2 × 2 × 2 mm3), and p-value are listed. To identify the activated areas, the Anatomical Automatic Labeling (AAL) atlas was used. FWE: family-wise error.

Supplementary Table 2 Correlation between brain activity and reliability

| Contrast | L.OFC | R.OFC | L.NAc | R.NAc | PCC |
| --- | --- | --- | --- | --- | --- |
| Sincere praise - flattery: fast | 0.13 | 0.06 | 0.16 | 0.24 | 0.21 |
| Sincere praise - flattery: slow | –0.12 | –0.07 | 0.18 | 0.05 | **0.50**** |

Supplementary Table 2 shows the Pearson's correlation coefficients between activity in five regions of interest and perceived reliability of praise (difference between sincere and flattery face avatars). NAc: nucleus accumbens; OFC: orbitofrontal cortices; PCC: posterior cingulate cortex. (*: Bonferroni-corrected p-value < 0.05; n = 5).

Supplementary Table 3 Correlation between brain activity and praise-seeking Supplementary

| Contrast | L.OFC | R.OFC | L.NAc | R.NAc | PCC |
| --- | --- | --- | --- | --- | --- |
| Sincere praise - flattery: overall | –0.03 | 0.03 | –0.06 | –0.28 | 0.05 |
| Sincere praise - flattery: fast | –0.02 | 0.08 | 0.18 | 0.08 | –0.09 |

Supplementary Table 3 shows the Pearson's correlation coefficients between activity in five regions of interest and the praise-seeking trait. NAc: nucleus accumbens; OFC: orbitofrontal cortex; PCC: posterior cingulate cortex. (†: uncorrected p-value < 0.05 for all speeds).

Supplementary Table 4 Correlation between activity in the left IPS and other personality traits

| Scale | Subscale | Subscale 2 | Pearson’s r |
| --- | --- | --- | --- |
| Big-Five personality inventory | Extraversion |  | 0.14 |
|  | Agreeableness |  | 0.2 |
|  | Conscientiousness |  | –0.47 † |
|  | Neuroticism |  | 0.40 † |
|  | Openness |  | 0.2 |
| Self-Esteem Scale |  |  | –0.34 |
| BIS/BAS | BIS |  | 0.43† |
|  | BAS | BAS | 0.34 |
|  |  | Drive | 0.04 |
|  |  | Fun seeking | 0.56* |
|  |  | Reward responsiveness | 0.17 |
| Daily Life Skills Scale | Positive thinking |  | –0.29 |
| Effortful control | Inhibitory control |  | –0.27 |
|  | Activation control |  | –0.43 † |
|  | Attentional control |  | –0.27 |

Supplementary Table 4 shows the Pearson's correlation coefficients between activity in the left IPS and other personality traits (comparison between the sincere-praise and control conditions; slow) (*: Bonferroni-corrected p-value < 0.05; n = 15; †: uncorrected p-value < 0.05)

Activity in the left IPS correlated with the fun-seeking BIS/BAS (behavioural approach system/ behavioural inhibition system) trait. This may be explained by high praise-seeking being reflected in the BIS/BAS (Kojima, 2018); therefore, BIS/BAS scores may be related to the brain areas activated by praise.

# Supplementary References

Carver, C. S., and White, T. L. (1994). Behavioral inhibition, behavioral activation, and affective responses to impending reward and punishment: The BIS/BAS scales. J. Pers. Soc. Psychol. 67, 319–333. doi: 10.1037/0022-3514.67.2.319.

Gosling, S. D., Rentfrow, P. J., and Swann, W. B. (2003). A very brief measure of the Big-Five personality domains. J. Res. Pers. 37, 504–528. doi: 10.1016/S0092-6566(03)00046-1.

Kojima, Y. (2018). A study of the relation temperament, praise-seeking need and rejection-avoidance need. Bull. Saitama Gakuen Univ. Fac. Humanit. 18, 51–58. Available online at: http://id.nii.ac.jp/1354/00001157/.

Oshio, A., Abe, S., and Cutrone, P. (2012). Development, reliability, and validity of the Japanese version of ten item personality inventory (TIPI -J). Japanese J. Personal. 21, 40–52.

Rosenberg, M. (1965). *Society and the adolescent self-image*. Princeton, NJ: Princeton University Press.

Rothbart, M. K., Evans, D. E., and Ahadi, S. A. (2000). Temperament and personality: Origins and outcomes. J. Pers. Soc. Psychol. 78, 122–135. doi: 10.1037/0022-3514.78.1.122.

Shimamoto, K., and Motonobu, I. (2006). Development of a daily life skills scale for college students. Japanese J. Educ. Psychol. 54, 211–221. doi: 10.5926/jjep1953.54.2_211

Takahashi, Y., Yamagata, S., Kijima, N., Shigemasu, K., Ono, Y., and Ando, J. (2007). Gray’s temperament model: Development of Japanese version of BIS/BAS scales and a behavior genetic investigation using the twin method. Japanese J. Personal. 15, 276–289. doi: 10.2132/personality.15.276.

Yamagata, S., Takahashi, Y., Shigemasu, K., Ono, Y., and Kijima, N. (2005). Development and validation of Japanese version of effortful control scale for adults. Japanese J. Personal. 14, 30–41. doi: 10.2132/personality.14.30

Yamamoto, M., Matsui, Y., and Yamanari, Y. (1982). The structure of perceived aspects of self. Japanese J. Educ. Psychol. 30, 64–68. doi: 10.5926/jjep1953.30.1_64.
